# Metal Ions Doping for Boosting Luminescence of Lanthanide-Doped Nanocrystals

**DOI:** 10.3389/fchem.2020.610481

**Published:** 2020-12-08

**Authors:** Shihao Pei, Xiaoqian Ge, Lining Sun

**Affiliations:** Research Center of Nano Science and Technology, College of Sciences, Shanghai University, Shanghai, China

**Keywords:** metal ion doping, host lattice manipulation, energy transfer modulation, lanthanide doped nanocrystals, enhanced luminescence

## Abstract

With the developing need for luminous materials with better performance, lanthanide-doped nanocrystals have been widely studied for their unique luminescence properties such as their narrow bandwidth emission, excellent chemical stability, and photostability, adjustable emission color, high signal-to-background ratio, deeper tissue penetration with less photo-damage, and low toxicity, etc., which triggered enthusiasm for research on the broad applications of lanthanide-doped nanocrystals in bioimaging, anti-counterfeiting, biosensing, and cancer diagnosis and treatment. Considerable progress has been made in the past few decades, but low upconversion luminescence efficiency has been a hindrance in achieving further progress. It is necessary to summarize the recently relevant literature and find solutions to improve the efficiency. The latest experimental and theoretical studies related to the deliberate design of rare earth luminescent nanocrystals have, however, shown the development of metal ion-doped approaches to enhance the luminescent intensity. Host lattice manipulation can enhance the luminescence through increasing the asymmetry, which improves the probability of electric dipole transition; and the energy transfer modulation offers a reduced cross-relaxation pathway to improve the efficiency of the energy transfer. Based on the mechanisms of host lattice manipulation and energy transfer modulation, a wide range of enhancements at all wavelengths or even within a particular wavelength have been accomplished with an enhancement of up to a hundred times. In this mini review, we present the strategy of metal ion-doped lanthanide nanocrystals to cope with the issue of enhancing luminescence, overview the advantages and tricky challenges in boosting the luminescence, and provide a potential trend of future study in this field.

## Introduction

Fluorescence imaging has attracted increasing attention for observing a vast number of biological structures due to high sensitivity, superior subcellular resolution, and ultrafast real-time imaging (Weissleder and Pittet, 2008). The frequent fluorescent probes that are exploited in imaging include fluorescent proteins (Ben et al., 2006), metal complexes (Zhao et al., 2010, 2011), organic fluorescent dyes (Terai and Nagano, 2008; Beija et al., 2009; Yuan et al., 2013), and semiconductor quantum dots (Zhou J. et al., 2015; Xu et al., 2016; Hildebrandt et al., 2017). However, most of them are excited by ultraviolet or visible light, which leads to significant background noise and low penetration depth (Fan and Zhang, 2019). Moreover, high-energy ultraviolet or visible light may cause cell apoptosis or tissue damage.

Alternatively, lanthanide-doped nanocrystals (LDNCs) are excited by near infrared (NIR) light, such as 980 or 808 nm, offering lower scattering coefficients and autofluorescence, and a higher penetration depth (Kobayashi et al., 2009; Yuan et al., 2013). Besides the NIR light excitation, the LDNCs also have several spectroscopic benefits: (1) a sharp emission band with a full-width at half-maximum (FWHM) <10 nm and a long decay lifetime (μs to ms) (Bünzli, 2010; Fan et al., 2018); (2) hundreds of nanometers of anti-Stokes or Stokes shift (upconversion or downshifting the luminescence process) (Su et al., 2017); and (3) excellent photo and chemical stability (no photoblinking or photobleaching) (Su et al., 2017). Although LDNCs possess such excellent spectroscopic characteristics, the major drawback of LDNCs is their low quantum yield (QY) due to the low extinction coefficient of lanthanide ions in the NIR region and energy lost during multi non-radiative electronic transitions (Wang et al., 2011). In recent years, researchers have been devoted to solving this drawback, such as constructing core-shell structures (Chen et al., 2015; Zhuo et al., 2017) and anchoring NIR dyes on the surface of nanocrystals (Wu X. et al., 2016; Hazra et al., 2018). Among countless methods for improving the QY (Zhang et al., 2010; Yin et al., 2016), metal ion doping is the simplest since it is carried out in fewer modulation steps (Niu et al., 2012; Ding et al., 2015), and it only weakly changes the shape of the LDNCs. Moreover, metal ion doping can integrate with other methods to simultaneously improve the QY of LDNCs.

Here, we aim to provide a summary regarding the recent progress in metal ion doping for improving the QY of LDNCs. In this mini review, we first discuss the two mechanisms of metal ion doping: host lattice manipulation and energy transfer modulation. More cases outlining how to carry out metal ion doping are also included. Finally, we discuss the challenges and future applications of LDNCs with metal ion doping. We also hope that this mini review can serve as a guide for researchers who are involved in metal ion doping for LDNCs study.

## Mechanisms of Luminescence Enhancement by Doping Metal Ions

### Host Lattice Manipulation

The optical characteristic of lanthanide NPs is derived from its intrinsic trivalent lanthanide ions (Ln^3+^), which are considered the most stable state of lanthanides. Ln^3+^ ions have the configuration of [Xe]4f^n^, *n* = 0–14, and the electronic transitions in the 4f orbital are diverse, resulting in the emissions from these electronic transitions being distributed within wide wavelengths (Hatanaka and Yabushita, 2014). There are several decisive factors for the energy levels of free Ln^3+^ ions in their 4f orbitals, like the Coulombic interaction and the spin-orbit coupling between f electrons (Han et al., 2014), which are rather sensitive to minor changes of the host lattice.

As for metal ion doping, the lanthanide ions sites in the host lattice are replaced, and the host lattice may be distorted and its interplanar spacing will change due to the radius difference between the metal ions and lanthanide ions. When using a high metal ion concentration, the lattice gap will be filled with metal ions (Dou and Zhang, 2011). Thus, the asymmetric host lattice affects the environment of inner lanthanide ions, leading to the increase of lanthanide luminescence. For example, the probability of the electric dipole transition can be dramatically increased by the asymmetric crystal field.

By disrupting the symmetric environment of the central ions, the mixing of the opposite-parity configurations can break the Laporte selection rule (Harris and Bertolucci, 1978), which was applied to a centrosymmetric system where the electric dipole transitions are barely allowed. Therefore, reasonably, with the introduction of the asymmetric crystal field, the probability of the electric dipole transition can be dramatically increased. Then the luminescence intensity of the nanocrystal can be enhanced by increasing the asymmetry around the lanthanides.

**Figure 1:**
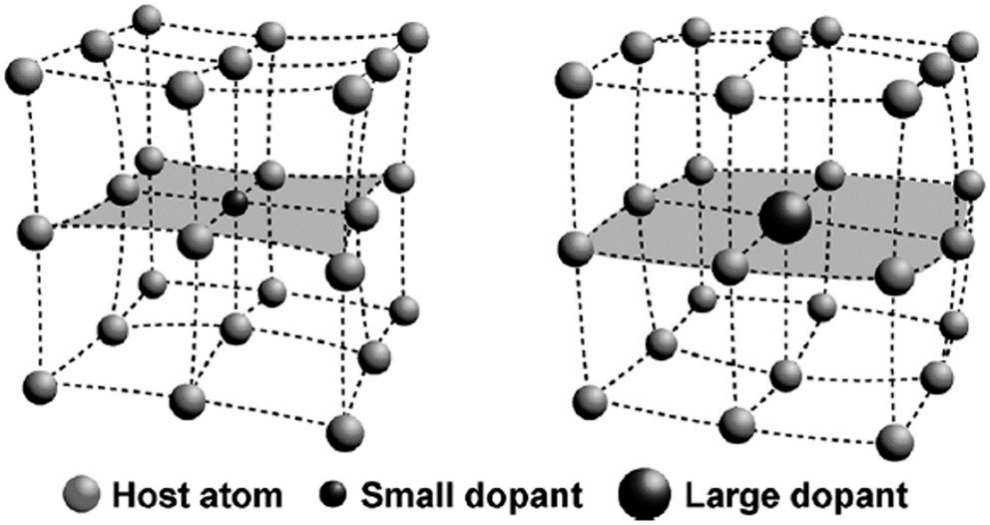
The contraction (left) and expansion (right) of the host lattice after adopting a small or large dopant. This figure was adopted from Han et al. (2014).

For doping metal ions with different radii, the host lattice may undergo different changes. The host lattice will shrink after doping metal ions with a small radius, whereas metal ions with a large radius lead to the expansion of the host lattice (Figure 1). The changing of the host lattice dramatically alters the splitting of the crystal field and the coordination environment, resulting in the increase of the probability of the electric dipole transition, and then enhancing the luminescence intensity (Han et al., 2014).

### Energy Transfer Modulation

All fluorescence light usually follows the well-known Stokes' law which illustrates that the energy level of the excitation photons is higher than the emitted photons, whereas the doping of lanthanides or transition metal ions can violate this principle under a properly powered excitation, generating anti-Stokes emission.

The anti-Stokes process is a multi-ions process. In principle, the premise of energy transfer is that the absorption and emission are not in the same center, and it can take place without charge transport. Moreover, energy transfer can be divided into radiative, non-radiative, resonant, and phonon-assisted energy transfer (Hatanaka and Yabushita, 2014). The energy transfer process contains two steps, whose natural efficiencies are ≤1. Therefore, finding a way to reduce the unwanted cross-relaxation type of energy transfer and converting this energy into a certain wavelength of emission improves the luminescent intensity by energy transfer modulation. Higher doping of activator ions could be a potential candidate that can lead to the enhancement of luminescence, whereas the quenching effect has been a hindrance in achieving this goal. Therefore, owing to the macroscopic diffusion process, the overall efficiency enhancement brought about by energy transfer can only be obtained through spatial averaging (Auzel, 2004).

The metal ion doping in the LDNCs can modulate the energy transfer between the doped metal ions and other lanthanide ions in the host lattice (Han et al., 2014). This modulation depends on controlling the re-distribution of all ions in the host lattice (Auzel, 2004). Specifically, the distance between activators and sensitizers in the host lattice is changed by doping metal ions, neither too long nor too short, which is crucial for boosting the luminescence intensity. For example, the increase of doping ions can facilitate the harmful cross-relaxation between dopants due to their proximity (Qin et al., 2019). For another method of manipulating the ion distribution reported by Qin et al. (2014), they indicate that, in a particular host lattice, the lanthanide ions tend to segregate in the form of chains or clusters upon host cation substitution. As a result, the five-photon upconversion (Wang et al., 2014) and single band emission (Wu M. et al., 2016) can be achieved.

Moreover, the re-distribution of activators decreases the probability of cross-relaxation, and a photon energy depletion pathway arises from activators or between an activator and a defect in the host lattice. Thus, the lower cross-relaxation probability allows us to use a higher activator concentration so that the luminescence intensity enhances, which is also called breaking the concentration quenching effect (Auzel, 2004). Most importantly, the d-d electronic transition of metal ions may be involved in the energy transfer between activators and sensitizers in the host lattice, and it is an effective energy transfer pathway (Han et al., 2014). So, if the energy transfer pathway produced by the doped transition metal ions works out, the upconversion luminescence of the LDNCs should be enhanced.

Unlike the normal f-f transition, the emission intensity of the hypersensitive transition will change dramatically even if its surrounding environment has a tiny change. By changing the environment around the rare-earth ions, a hypersensitive transition can be produced. The doping of different kinds of metal ions should be an easy way to alter the environment around the rare-earth ions. It is well-known that the upconversion luminescence intensity of lanthanide ions is mainly dependent on electronic transition probabilities (Hatanaka and Yabushita, 2014). Owing to their unique properties, increasing the probability of hypersensitive transition will be beneficial in increasing the luminescence intensity of LDNCs.

## Cases of Metal Ion-doped Lanthanide Nanocrystals and Their Bioapplications

### Li^+^ Ion Doping

**Figure 2:**
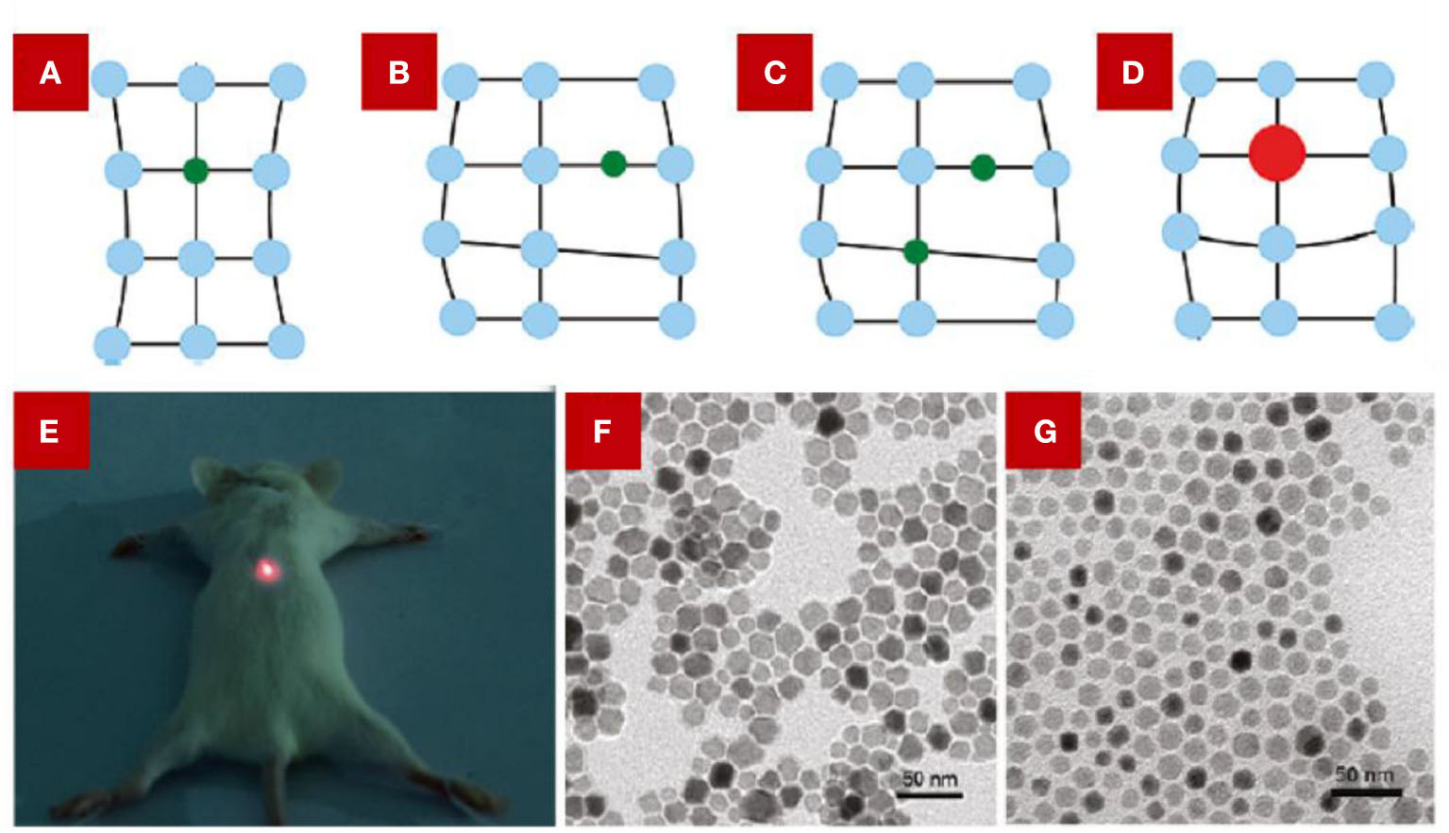
The scheme shows the possible ways of doping alkali ions in the host lattice of NaYF_4_. **(A)** Substitution by a small atom. **(B)** Interstitial occupation by a small atom. **(C)** Combination of substitution and interstitial occupation. **(D)** Substitution by a large atom. From Dou and Zhang (2011). **(E)** The digital image of an anesthetized mouse under the irradiation of a 980 nm laser. The mouse was injected the GdF_3_:Er,Yb co-doped with Li^+^ dispersion in the back muscle. **(E)** was adopted from Yin et al. (2012). And **(F,G)** are TEM images of Na_(1−x)_Li_x_YF_4_:Er,Yb NCs. **(F)** x = 40 mol%, **(G)** x = 60 mol%. **(F,G)** were adopted from Dou and Zhang (2011).

The radius of metal ions has a significant impact on the luminescent intensity of LDNCs through changing the symmetry of its host lattice around the lanthanide ions (Figures 2A–D). This impact was verified by doping ions, including Li^+^, Ca^2+^ (Zhao et al., 2020), and Bi^3+^ ions (Jiang et al., 2012; Niu et al., 2012) into the host lattice. For example, the Li^+^ ion owns the smallest alkali ionic radius, around 0.73–1.06 Å, enabling a high doped concentration in the host lattice (Dou and Zhang, 2011). Chen et al. reported that 5% of Li^+^-doped Y_2_O_3_:Yb,Er nanocrystals (NCs) (Chen et al., 2008) show 25 times and 8 times more luminescent intensity enhancements for the green and red emissions, respectively, in comparison with Y_2_O_3_:Yb,Er NCs without Li^+^ doping. The similar luminescence enhancement was also found in Li^+^-doped ZrO_2_ (Liu et al., 2011), and NaGd(MoO_4_)_2_ (Chen et al., 2020) host lattices. However, the symmetry of the host lattice may undergo different changes when using different synthesized temperatures. For example, at 1,073 K, the Er^3+^-Li^+^ co-doped TiO_2_ NCs showed an accelerated phase transition from anatase to rutile when increasing the Li^+^ concentration, resulting in the decrease of luminescence intensity. At 1,273 K, the phase structure of Er^3+^-Li^+^ co-doped TiO_2_ NCs was unchanged no matter what the Li^+^ concentration was, yet the crystal field symmetry decreased, resulting in significantly enhanced emission intensities (Cao et al., 2010).

Besides, it was reported that the doping of Li^+^ can achieve the enhancement of luminescent intensity at a particular wavelength range. Yin et al. (2012) reported that GdF_3_:Er,Yb co-doped with Li^+^, with a color-tuned emission from yellow to red, showed a slightly decreased green emission whereas the red emission had a dramatic increase of up to 8-fold. And the red light displayed a deep penetration depth, which was used for the *in vivo* imaging (Figure 2E). This phenomenon can be attributed to the energy back transfer process initiated by the doping of Li^+^ ions.

As for host lattice materials, a fluoride-based host lattice is an excellent candidate for co-doped Li^+^ ions. In 2017, Hu et al. (2017) reported 18 times and 7 times luminescence enhancement of 478 and 804 nm emissions of NaLuF_4_:Yb,Tm with a 7% Li^+^-doped concentration, respectively. Zhao C. et al. (2013) developed 8 times luminescence enhancement of the upconversion emission of 452 nm in NaYF_4_:Yb,Tm NCs with a Li^+^ concentration of 7%. Furthermore, Ding et al. (2015) studied different kinds of lanthanide ions co-doped with Li^+^ in an NaGdF_4_ host lattice crystal, which all afford large enhancement in lanthanide luminescence intensity. Dou and Zhang (2011) have concluded the possible substitution sites of Li^+^-doped and K^+^-doped ions in an NaYF_4_ host lattice, respectively, in which the substitution and the interstitial occupation both exist, only related to the concentration of the doping ion. As shown in Figures 2F,G, with an increase in Li^+^ concentration from 40 to 60%, the morphology of nanocrystals changed from nanorods to nanospheres, and the phase changed from a hexagonal to a cubic phase as well.

What also needs to be considered is the doping efficiency. Wang et al. studied the doping efficiency of Li^+^ in the KSc_2_F_7_ host lattice, they indicated that Li^+^ doping efficiency is highly related to its initial concentration (Wang et al., 2017). In general, the actual amount of Li^+^ doped into the host lattice is much lower than its initial concentration.

### Fe^3+^ ion Doping

**Figure 3:**
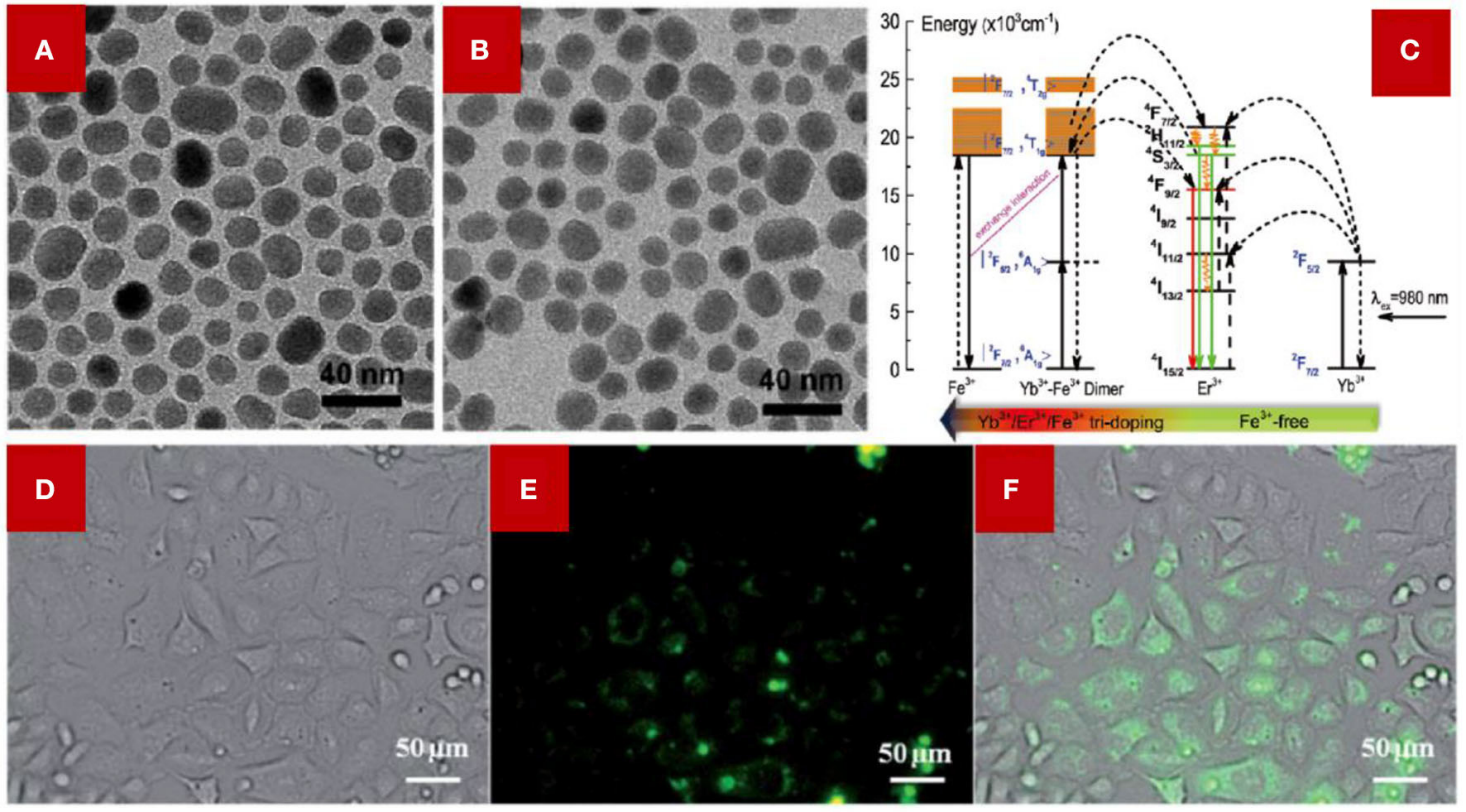
TEM images of NaGdF_4_:Yb,Er,Fe NCs, **(A)** 0 mol % Fe^3+^ doping; **(B)** 30 mol% Fe^3+^. Adopted from Ramasamy et al. (2013) **(C)** Illustration of the proposed energy transfer mechanism of Fe^3+^ co-doped NaYF_4_:Yb,Er NCs and NaYF_4_:Yb,Er NCs without Fe^3+^ doped. The color arrow in the below indicates the Fe^3+^ content varied from 0 to 5–40 mol%. **(C)** was adopted from Tang et al. (2015); **(D–F)** NaGdF_4_:Yb,Er,Fe NCs were used for cellular luminescence imaging. **(D)** The bright field image of HeLa cells incubated with NaGdF_4_:Yb,Er,Fe NCs. **(E)** The confocal fluorescence image. The HeLa cells were irradiated with a 980 nm laser. **(F)** The merging of **(D,E)**. **(D,E)** were adopted from Ramasamy et al. (2013).

Similar to Li^+^, Fe^3+^ doping can also alter the symmetry of the host lattice in LDNCs. In an NaGdF_4_ host lattice, the Fe^3+^ doping can meet the goal of boosting the luminescent intensity through altering the asymmetry around the lanthanide ions, and this enhancement is a general improvement for all emission ranges around the luminescence center (Ramasamy et al., 2013). Interestingly, the synthesis method of LDNCs influences the crystal structure, and thus affects the symmetry of the host lattice after doping Fe^3+^. For example, the hexagonal NaYF_4_ became tetragonal after increasing the Fe^3+^ concentration when using a hydrothermal method (Tang et al., 2015). However, the crystal structure of the NaGdF_4_ host lattice is inert to the thermal decomposition even using a high Fe^3+^ concentration (Figures 3A,B) (Ramasamy et al., 2013). In addition, the doping of Fe^3+^ can tailor the crystal field environment of Er^3+^, which helps the hypersensitive transition, leading to the enhancement of upconversion luminescence (Ramasamy et al., 2013).

Besides, the Yb^3+^-Fe^3+^ dimer was formed in the host lattice, which can be applied to modulate the energy transfer between activators and sensitizers, in particular modulating the energy transfer for the red emission (Tang et al., 2015; Du et al., 2019). For the Fe^3+^-doped NaYF_4_:Yb,Er NCs (Figure 3C), the existence of a Yb^3+^-Fe^3+^ dimer can be deduced from the much lower *n* value (*n* represents the number of phonons process) of green/red emissions of the hydrothermal method-synthesized LDNCs compared with the traditional two-phonon process. The energy level of |^2^F_7/2_, ^4^T_1g_ > of the Yb^3+^-Fe^3+^ dimer receives the photon energy from the energy level of ^2^H_11/2_ of Er^3+^, and the received photon energy can partially return to the energy level of ^4^F_7/2_ and ^4^S_3/2_ of Er^3+^. After the photon energy in ^4^F_7/2_ and ^4^S_3/2_ relaxes to the ^4^F_9/2_ level, the probability of the electronic transition between ^4^F_9/2_ and ^4^I_15/2_ of Er^3+^ increases, which contributes to increase the intensity of the red emission of Er^3+^. Meanwhile, the green/red emission of Fe^3+^-doped NCs synthesized by the thermal decomposition method has been confirmed as a two-phonon process (Ramasamy et al., 2013), which has less correlation with the Fe^3+^ concentration. However, the Fe^3+^-doped NCs synthesized by the hydrothermal method show the potential relationship between the concentration of Fe^3+^ and the phonon process, suggesting that the formation of the Yb^3+^-Fe^3+^ dimer might be related to the synthesis method of the LDNCs. Furthermore, the Fe^3+^-doped NCs were used in the upconversion luminescence (UCL) imaging of HeLa cells (Figures 3D–F), which demonstrates a potential application in bioimaging.

### Mn^2+^ ion Doping

**Figure 4:**
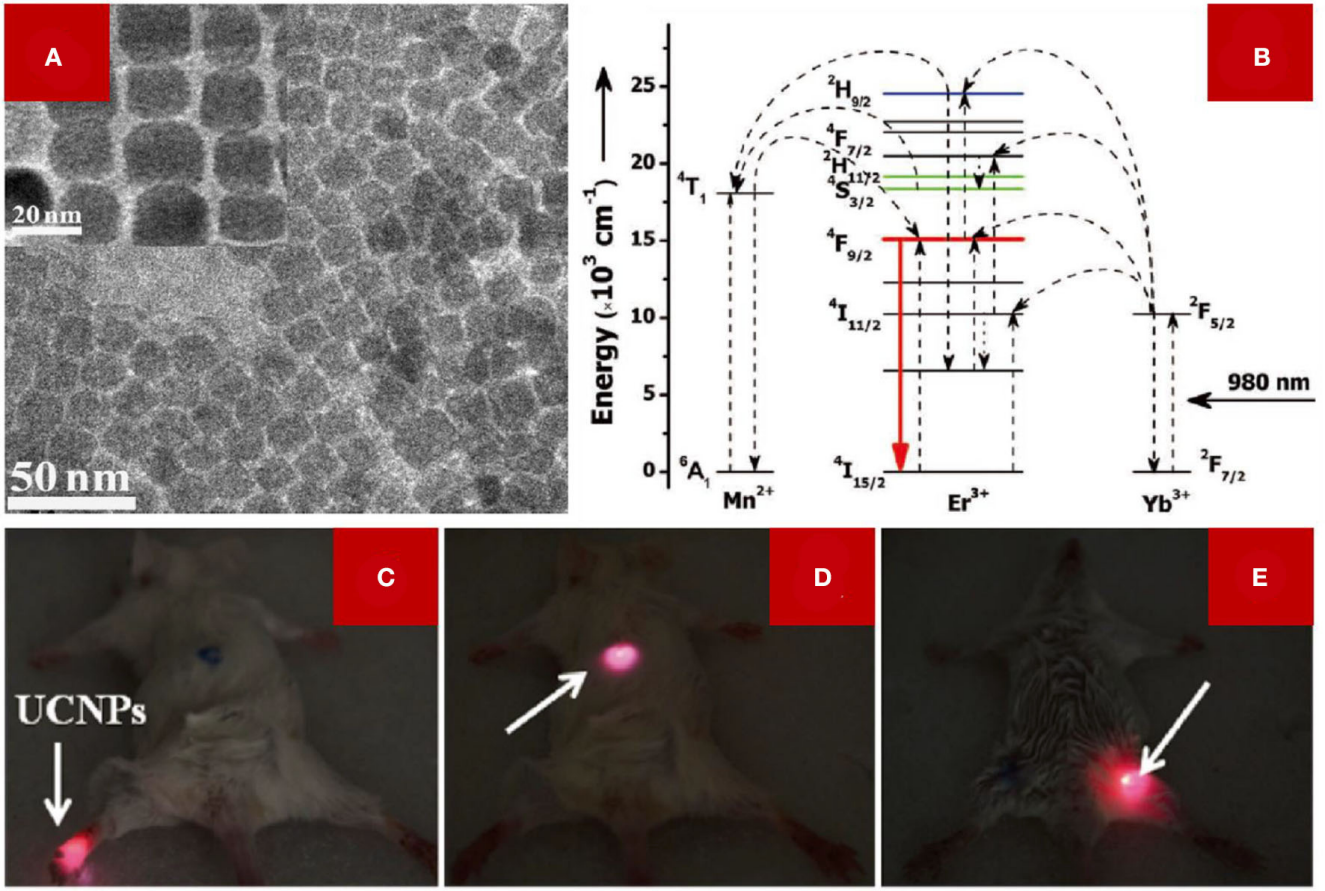
**(A)** The TEM image (inset: HRTEM image) of NaYF_4_:Er,Yb NCs doped with 30 mol% Mn^2+^ ions. **(B)** Schematic illustration of the mechanism of single red emission of Mn^2+^-doped NaYF_4_: Yb/Er NCs. **(C–E)** Small animal imaging of Kunming mouse with irradiation of a 980 nm laser. The arrow indicates the injected site of the LDNCs dispersion. **(A–E)** were adopted from Tian et al. (2012).

It was discovered that the Mn^2+^ ion can also modulate the energy transfer between activators and sensitizers in the host lattice of LDNCs (Wang J. et al., 2011). With the example of Mn^2+^-doped NaYF_4_:Yb/Er NCs (Figures 4A,B), the single red emission can be ascribed to the energy transfer from the ^2^H_9/2_ and ^4^S_3/2_ energy level of Er^3+^ to the ^4^T_1_ energy level of Mn^2+^, and then the received photon energy in ^4^T_1_ transfers back to the ^4^F_9/2_ energy level of Er^3+^, which contributes to the red emission of the NaYF_4_:Yb/Er NCs (^4^F_9/2_→^4^I_15/2_) (Tian et al., 2012). The single red emission phenomenon was also confirmed in the Mn^2+^-doped NaYF_4_ (Zeng et al., 2014), NaGdF_4_ (Li et al., 2015), and NaLuF_4_ (Zeng et al., 2014) host lattices.

Owing to the sensitivity of Mn^2+^ to the ligand field (Zhou et al., 2018), the energy level state of ^4^T_1_ is different in different host lattices, such as NaGdF_4_ or LiYF_4_. The higher energy level of ^4^T_1_ of Mn^2+^ in an LiYF_4_ host lattice matches the green luminescence energy level of ^4^S_3/2_ of Er^3+^, and the lower energy level of ^4^T_1_ of Mn^2+^ matches better with the red emission energy level of ^4^F_9/2_ of Er^3+^ in the NaGdF_4_ host lattice, and thus explains that the ratio of green and red emission intensity is not a fixed value (Zhou et al., 2018). Thus, the sensitivity of Mn^2+^ endorses the potential application of Mn^2+^ in modulating the energy transfer of different lanthanide ions. Moreover, Mn^2+^ doping can induce the phase transition of the NaYF_4_ host lattice, from hexagonal to cubic, when using the hydrothermal method. In spite of the fact that the cubic NaYF_4_ lattice was confirmed to have a lower luminescence efficiency than the hexagonal one, the asymmetry of the NaYF_4_ changed because the Y^3+^ sites were replaced by Mn^2+^ ions with a smaller radius, then the luminescence intensity increased. Fig. 4a shows the cubic phase of NaYF_4_:Yb,Er doped with 30 mol% Mn^2+^ ions. The red emission has a higher signal-to-noise ratio and lower autofluorescence than the green emission. Therefore, lanthanide NCs with a single red emission are beneficial for *in vivo* small-animal imaging (Figures 4C–E).

## Rare Earth Ions Doping

Besides activators and sensitizers, doping other rare earth ions into a host lattice is also an efficient way to boost the luminescence intensity. For example, Sc^3+^ has been widely studied because it has the smallest radius among all the rare earth ions, meaning it can be easily doped into a host lattice. With a similar host lattice manipulation mechanism, the symmetry of an NaYF_4_ host lattice was broken when doping Sc^3+^ into NaYF_4_:Er,Yb NCs; thus, the overall emission intensity of NaYF_4_:Er,Yb NCs showed a 2-fold enhancement when doped with 10% mol Sc^3+^ (Huang et al., 2010).

**Figure 5:**
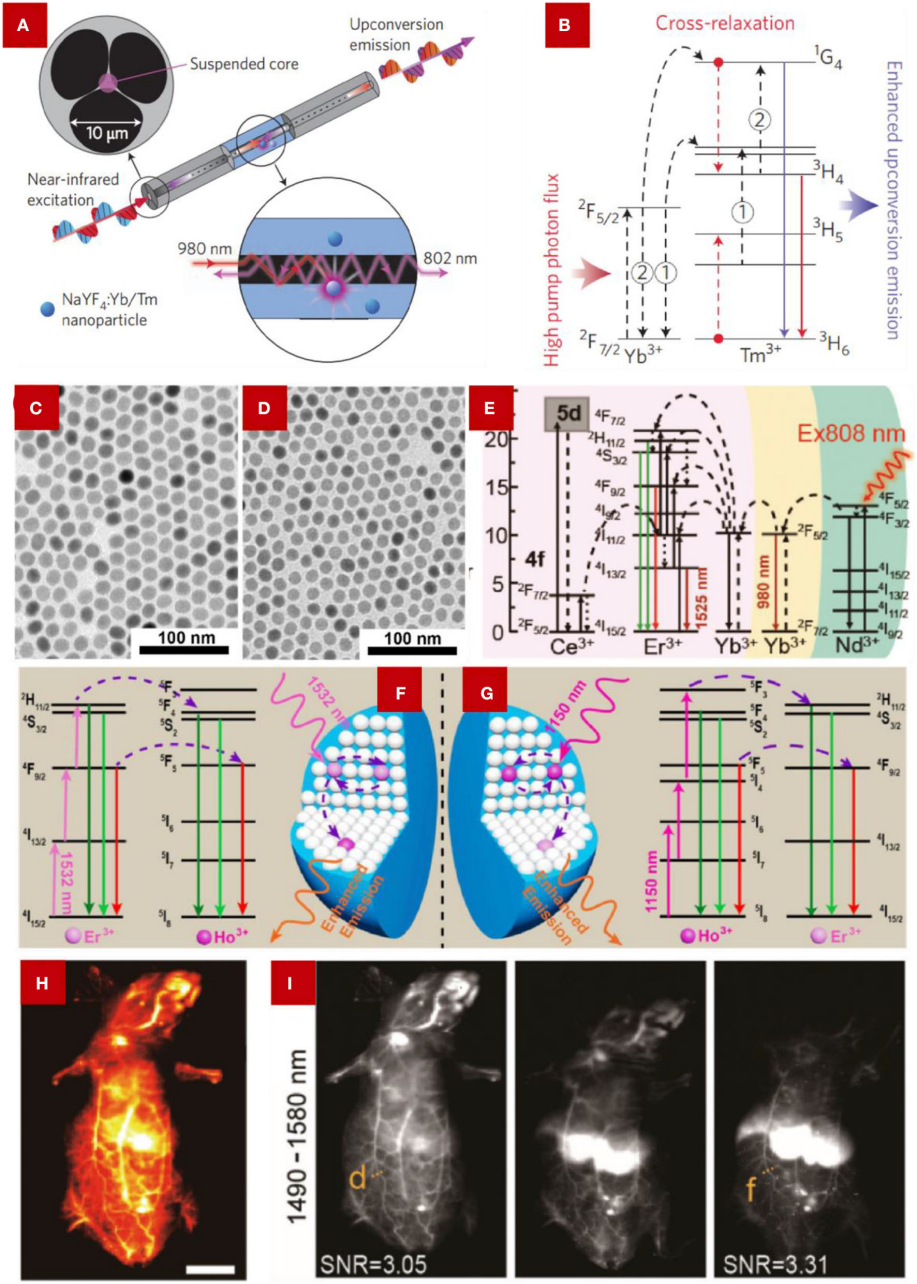
**(A)** Schematic illustration of the integrated system with the micro-structured optical fiber with NaYF_4_:Yb/Tm nanoparticles and **(B)** the simplified mechanism of the energy transfer between Yb^3+^ and Tm^3+^ under 980 nm excitation, 1 and 2 represent two subsequent energy-transfer processes from Yb^3+^ to Tm^3+^ ions. From Zhang and Liu (2013). TEM images of **(C)** NaYF_4_:Er/Ho (10/0.2 mol%) NCs, **(D)** NaYF_4_:Er/Ho (10/1 mol%) NCs. **(C,D)** were adopted from Cheng et al. (2018). **(E)** Schematic illustration of energy transfer in Ce^3+^-doped NaYbF_4_:Ce,Er NCs for boosting the ~1,525 nm emission. **(C)** were adopted from Cao et al. (2020). **(F,G)** Illustration of the scheme of the energy transfer process of Er^3+^ and Ho^3+^ under the irradiation of 1,532 and 1,150 nm, respectively. From Cheng et al. (2018). **(H)** Blood vessels imaging using NaYbF_4_:Er,Ce@NaYF_4_:Yb@NaYF_4_:Nd NCs. **(I)** NIR region luminescence collected from different time points (10 min, 4 h, 10 h). The emission light was collected between the 1,490–1,580 nm regions, with the excitation of an 808 nm laser. **(H,I)** were adopted from Cao et al. (2020).

And other lanthanide ions, for example, activator ions with a high doping concentration such as Er^3+^, Tm^3+^, and Ho^3+^, have been used to accept photon energy from sensitizer ions. However, the high concentration activator ions lead to the concentration quenching effect that is generated from cross-relaxation between activator ions. Zhao J. et al. (2013) built a combined system with micro-structured optical fiber (Figure 5A), which confines the high-power laser into a micrometer-sized circle. They demonstrated that a 70-fold enhanced luminescence intensity was obtained with high concentration doping of 8% mol Tm^3+^. This method solved the quenching effect using a high-power pump up to 2.5 10^6^ W/cm^2^. The quenching effect is mainly caused by the cross-relaxation of the ^1^G_4_ to ^3^H_4_ and ^3^H_6_ to ^3^H_5_ transitions, which usually occur under low-power irradiation (Figure 5B). For the high-power laser, the ^3^H_4_ energy level is more likely to be promoted to a higher level, which leads to the absence of cross-relaxation and the enhanced upconversion emission intensity as well. Other similar results were illustrated, like the high doping level of Yb^3+^, to enhance the luminescence of a sub 10 nm matrix, and the authors claimed that the enhanced upconversion emission intensity was endorsed by the energy transfer from Yb^3+^ to Tm^3+^ (Zhai et al., 2014).

In addition, the overlap between the energy levels of different rare earth ions was studied. Cheng et al. (2018) demonstrated an energy level overlap of Er^3+^ and Ho^3+^, which lead to enhanced upconversion luminescence. Figures 5C,D show the TEM images of different concentrations of Ho^3+^-doped NaYF_4_:Yb,Er. Because of the similar ion sizes of Er^3+^ and Ho^3+^, the host lattice manipulation should be excluded. Under excitation at 1,532 nm (the energy can only be absorbed by Er^3+^), part of the energy is accumulated at the ^2^H_11/2_ and ^4^F_9/2_ energy levels of the over-doped Er^3+^ ion, then the energy could be transferred to the ^5^F_4_/^5^S_2_ and ^5^F_5_ energy levels of the co-doped Ho^3+^ ions. Then the emissions at 544/550 and 648 nm, which are produced by Ho^3+^, overlapped with the emissions of Er^3+^ at 525/545 and 660 nm (Figures 5F,G). Therefore, the enhanced upconversion emissions of Er^3+^ and Ho^3+^ co-doped nanocrystals can be ascribed to the dual contribution from both Ho^3+^ and Er^3+^ ions.

As for the downshifting luminescence of lanthanide ions, the gadolinium-based host lattice has been widely studied due to its optically active Gd^3+^ sublattices (Wang F. et al., 2007). The energy transfer between Ce^3+^ and Ln^3+^ (Ln = Tb, Eu, Sm, or Dy) can be quickly achieved by a Gd^3+^ sublattice over a long distance. One of the benefits that the Gd^3+^ sublattice affords is that migrating energy can be trapped at a rather low doping concentration. Also, Gd^3+^ can act as an intermediary state to solve electron transfer quenching like the energy transfer from Ce^3+^ to Eu^3+^ (Wang F. et al., 2007). Ce^3+^ can also promote the energy transfer between activators and sensitizers. In 2019, Li et al. (2019) reported an enhanced downshifting emission located at ~1,525 nm by doping Ce^3+^ into NaLuF_4_:Gd/Yb/Er nanorods. The ~1,525 nm second near-infrared (NIR-II) emission was produced from the electronic transition between the excited state ^4^I_13/2_ to ^4^I_15/2_ of Er^3+^. However, the energy gap between the ground state ^2^F_5/2_ and the excited state ^2^F_7/2_ of Ce^3+^ matches well with the energy difference between the electronic transition of ^4^I_11/2_→^4^I_13/2_ of Er^3+^. The excited state ^4^I_11/2_ of Er^3+^ could suffer an efficient non-radiative phonon-assisted cross-relaxation process, resulting in the significantly accumulated excited state ^4^I_13/2_ of Er^3+^. Therefore, the electronic transition of ^4^I_13/2_→^4^I_15/2_ of Er^3+^ is also promoted, which helps enhance the ~1,525 nm emission intensity (Figure 5E). Cao et al. (2020) reported a similar result when they synthesized a core-shell structure of NCs, NaYbF_4_:Er,Ce@NaYF_4_:Yb@NaYF_4_:Nd. Under 808 nm excitation, the NaYbF_4_:Er,Ce@NaYF_4_:Yb@NaYF_4_:Nd NCs showed a 10 times luminescence enhancement at 1525 nm than the NCs without doping Ce^3+^. As the light in the NIR-II window affords a superior signal to noise ratio and lower autofluorescence than the light in the first NIR window, the NIR-II emission gets increasing attention in deep tissue imaging. In Figures 5H,I, the blood vessels can be clearly observed by using the NaYbF_4_:Er,Ce@NaYF_4_:Yb@NaYF_4_:Nd NCs, and the resolution can remain up to 0.25 mm even 10 h after the injection. Therefore, these excellent properties provide a potential application for LDNCs in the bioimaging fields.

## Summery and Perspectives

Through summarizing the recent studies on how to improve the luminescence of LDNCs, breakthroughs have been made in some aspects. In this review, we summarize the recently reported methods on how to boost the upconversion and even downshift luminescence by doping metal ions, and introduce the related two mechanisms of doping in detail (Table 1).

**Table 1:** The summary of doping different metal ions into suitable host lattices and their possible mechanism for enhanced luminescence.

**Metal ions**	**Host lattice**	**Crystal phase**	**Synthesized methods**	**Possible mechanism**
Li^+^	ZrO_2_ (Liu et al., 2011)	Monoclinic phase Tetragonal phase	Sol-gel process	Host lattice manipulation
	BaTiO_3_ (Sun et al., 2011)	Cubic phase	Sol-gel process	
	Y_2_O_3_ (Chen et al., 2008)	Cubic phase	–	
	NaYF_4_ (Dou and Zhang, 2011; Zhao C. et al., 2013)	Hexagonal phase	High temperature thermal-decomposition method	
	NaGdF_4_ (Ding et al., 2015)	Hexagonal phase	Co-precipitation method	
	NaLuF_4_ (Hu et al., 2017)	Hexagonal phase	Solvothermal method	
	TiO_2_ (Cao et al., 2010)	Anatase phase Rutile phase	Sol-gel process	
	GdF_3_ (Yin et al., 2012)	Orthorhombic phase	Hydrothermal procedure	
Fe^3+^	NaBiF_4_ (Du et al., 2019)	Hexagonal phase	Chemical precipitation method	Host lattice manipulation, energy transfer modulation
	NaGdF_4_ (Ramasamy et al., 2013)	Hexagonal phase	High temperature thermal-decomposition method	
	NaYF_4_ (Tang et al., 2015)	Hexagonal phase Cubic phase	Hydrothermal method	
Mn^2+^	NaYF_4_ (Tian et al., 2012; Zeng et al., 2014)	Hexagonal phase Cubic phase	Solvothermal method	Host lattice manipulation, energy transfer modulation
	NaLuF_4_ (Zeng et al., 2014)	Hexagonal phase Cubic phase	Hydrothermal method	
	NaGdF_4_ (Li et al., 2015)	Cubic phase	Thermal decomposition	
	NaYbF_4_ (Zeng et al., 2014)	Hexagonal phase Cubic phase	Hydrothermal method	
	NaMnF_3_ (Zhang et al., 2012)	Cubic phase	High temperature thermal-decomposition method	
	KMnF_3_ (Ning et al., 2020)	Cubic phase	High temperature thermal-decomposition method	
	LiYF_4_ (Zhou et al., 2018)	Tetragonal phase	Thermal decomposition	
Zn^2+^	NaYbF_4_ (Zhong et al., 2019)	Cubic phase	High temperature thermal-decomposition method	Host lattice manipulation
Ca^2+^	NaYF_4_ (Zhao et al., 2020)	Cubic phase Hexagonal phase	Co-precipitation method	Host lattice manipulation
Bi^3+^	NaYF_4_ (Niu et al., 2012)	Cubic phase Hexagonal phase	Facial microwave reflux method	Host lattice manipulation
Ce^3+^	NaYF_4_ (Li et al., 2019)	Hexagonal phase	Hydrothermal process	Energy transfer modulation
	NaYbF_4_ (Zhong et al., 2017; Li et al., 2019; Cao et al., 2020)	Cubic phase	Thermolysis method	
	NaLnF_4_ (Li et al., 2019)	Hexagonal phase	Hydrothermal process	
	NaGdF_4_ (Wang F. et al., 2007; Li et al., 2019)	Hexagonal phase	Hydrothermal process	
Eu^3+^	NaErF_4_ (Shang et al., 2018)	Hexagonal phase	High-temperature co-precipitation method	Energy transfer modulation
	NaGd(MoO_4_)_2_ (Chen et al., 2020)	Tetragonal phase	Solid-state reaction method	
Tb^3+^	NaYbF_4_ (Zhou B. et al., 2015)	Hexagonal phase	Co-precipitation method	Energy transfer modulation
Ho^3+^	NaYF_4_ (Cheng et al., 2018)	Hexagonal phase	High-temperature co-precipitation method	Energy transfer modulation
	LiYF_4_ (Cheng et al., 2018)	Hexagonal phase	High-temperature co-precipitation method	
Sc^3+^	NaYF_4_ (Huang et al., 2010)	Hexagonal phase	Hydrothermal method	Energy transfer modulation
Tm^3+^	NaErF_4_ (Chen et al., 2017; Shang et al., 2018; Zhang et al., 2019)	Hexagonal phase	High-temperature co-precipitation method	Energy transfer modulation

Through the manipulation of the host lattice, the metal ions play a significant role in increasing the asymmetry around the lanthanides, therefore, leading to the increase of dipole transition. And for the energy transfer modulation, metal ions improve the overall energy transfer efficiency and decrease the quenching effect by providing an effective pathway in the process of energy transfer.

On the basis of improving emission intensity, some problems, still have not been explained clearly. For example, when introducing metal ions into the main lattice, especially for the mechanism of energy transfer regulation, the influence of the host lattice manipulation should not be ignored, which may disrupt the accuracy of the results. To resolve this effect, it can only be reduced by introducing metal ions with a small difference in the ion radius between the doping and occupied ions. And for ions like Fe^3+^, the enhancement of luminescence is the result of the combination of the two mechanisms. At the same time, how the metal ion is doped into the host lattice has not been fully explained. Site occupation or lattice filling might both exist in the same host lattice, and the method of metal ion doping has a relatively close relationship with the concentration and radius of the doping ions.

With further exploration of the research, the importance of the NIR-II light also shows itself. For deeper penetration, higher signal-to-noise ratio, and smaller scattering effect, the NIR-II light arouses increasing attention. Therefore, a rapidly growing number of recent studies have focused on improving near infrared luminescence, such as the emission of Er^3+^ at ~1,530 nm. The application of Er^3+^ in biological imaging is worth exploring. In addition, the enhancement of a certain emission band which was caused by non-radiative transition between two or more different rare earth ions is also the main focus of future research. It is necessary to develop more non-radiative transitions between different rare earth ions with good energy level matching to enhance the desired emission intensity. For now, most studies on the enhanced luminescence have been applied in the biological field, such as bioimaging and biosensing. There are some other applications that have been explored for the broader use of enhanced luminescence, such as anti-counterfeiting (Ding et al., 2020) and finger print latency (Wang et al., 2020), which endorse the importance of enhancing luminescence. Nonetheless, we believe that the rare earth ion-doped upconversion nanomaterials will have a wider range of applications and bright prospects in basic studies and technology fields.

## Author Contributions

SP collected and read papers, and wrote the draft manuscript. XG and LS outlined the main text content, discussed, and revised the manuscript. All authors contributed to the article and approved the submitted version.

## Conflict of Interest

The authors declare that the research was conducted in the absence of any commercial or financial relationships that could be construed as a potential conflict of interest.
